# Racial differences in treatment and survival in older patients with diffuse large B-cell lymphoma (DLBCL)

**DOI:** 10.1186/1471-2407-10-625

**Published:** 2010-11-12

**Authors:** Robert Griffiths, Michelle Gleeson, Kevin Knopf, Mark Danese

**Affiliations:** 1 Outcomes Insights, Inc., Westlake Village, CA, USA; 2 Johns Hopkins University School of Medicine, Baltimore, MD, USA; 3 California Pacific Medical Center, San Francisco, CA, USA

## Abstract

**Background:**

Diffuse large B-cell lymphoma (DLBCL) comprises 31% of lymphomas in the United States. Although it is an aggressive type of lymphoma, 40% to 50% of patients are cured with treatment. The study objectives were to identify patient factors associated with treatment and survival in DLBCL.

**Methods:**

Using Surveillance, Epidemiology, and End Results (SEER) registry data linked to Medicare claims, we identified 7,048 patients diagnosed with DLBCL between January 1, 2001 and December 31, 2005. Patients were followed from diagnosis until the end of their claims history (maximum December 31, 2007) or death. Medicare claims were used to characterize the first infused chemo-immunotherapy (C-I therapy) regimen and to identify radiation. Multivariate analyses were performed to identify patient demographic, socioeconomic, and clinical factors associated with treatment and with survival. Outcomes variables in the survival analysis were all-cause mortality, non-Hodgkin's lymphoma (NHL) mortality, and other/unknown cause mortality.

**Results:**

Overall, 84% (n = 5,887) received C-I therapy or radiation treatment during the observation period: both, 26%; C-I therapy alone, 53%; and radiation alone, 5%. Median age at diagnosis was 77 years, 54% were female, 88% were white, and 43% had Stage III or IV disease at diagnosis. The median time to first treatment was 42 days, and 92% of these patients had received their first treatment by day 180 following diagnosis. In multivariate analysis, the treatment rate was significantly lower among patients ≥ 80 years old, blacks versus whites, those living in a census tract with ≥ 12% poverty, and extra-nodal disease. Blacks had a lower treatment rate overall (Hazard Ratio [HR] 0.77; P < 0.001), and were less likely to receive treatment within 180 days of diagnosis (Odds Ratio [OR] 0.63; P = 0.002) than whites. In multivariate survival analysis, black race was associated with higher all-cause mortality (HR 1.24; P = 0.01) and other/unknown cause mortality (HR 1.35; P = 0.01), but not mortality due to NHL (HR 1.16; P = 0.19).

**Conclusions:**

In elderly patients diagnosed with DLBCL, there are large differences in treatment access and survival between blacks and whites.

## Background

DLBCL is the most common type of NHL, with an estimated 20,300 new cases in the United States in 2010 [1-3]. It is classified as an aggressive form of NHL [3] because survival is limited in the absence of effective treatment [4]. There have been substantial changes in the treatment of DLBCL in the past two decades. For instance, prior to the introduction of the monoclonal antibody rituximab (Genentech, South San Francisco, CA), the mainstay of treatment for DLBCL was CHOP (cyclophosphamide, doxorubicin, vincristine, and prednisone), with a three year overall survival of approximately 54% [5]. In 2002, a landmark study by Coiffier et al. demonstrated that rituximab plus CHOP (R-CHOP) significantly improved overall survival compared to CHOP alone in elderly patients with DLBCL [6]. Based on this study, and later studies that confirmed the findings [7-10], R-CHOP is now recommended as frontline therapy for most patients with advanced (Ann Arbor Stage III-IV) disease and many with localized (Ann Arbor stage I-II) disease [3].

The existence of racial and ethnic disparities in health care access and outcomes is well-documented. An Institute of Medicine report [11] found that even when access-related factors such as insurance status and the ability to pay for care are the same, African Americans and Hispanics tend to receive a lower quality of health care across a range of disease areas and clinical services, including cancer. According to the American Cancer Society, African Americans are more likely to develop and die from cancer than any other racial or ethnic group [1]. Not surprisingly, therefore, considerable attention has been focused on better understanding racial and ethnic disparities in cancer care and outcomes, including NHL [12-14].

One recent study by Wang and colleagues examined ethnic variations in treatment and survival in patients diagnosed with NHL, including DLBCL and two indolent types of NHL: chronic lymphocytic leukemia (CLL); and follicular lymphoma (FL) [14]. This was a retrospective cohort study of patients diagnosed with NHL from 1992 to 1999, using the SEER-Medicare linked database. Based on multivariate analysis, the investigators found that among all patients diagnosed with NHL, blacks were significantly less likely than whites to receive chemotherapy (OR, 0.69; 95% confidence interval [CI], 0.50-0.95). The study found no difference in all-cause mortality between blacks and whites, either overall or in any of the specific types of NHL, including DLBCL.

Several changes in the treatment of NHL have occurred since the patients included in this study were diagnosed, including the introduction of rituximab, updated clinical practice guidelines, and ongoing pressures on the health system to reduce costs while improving outcomes. These may have impacted racial and ethnic disparities in access and outcomes in NHL, and in particular aggressive types such as DLBCL. For instance, the impact of racial and ethnic disparities in access to treatment for DLBCL, as documented by Wang and colleagues [14], could increase disparities in outcomes such as survival in the presence of more effective treatments such as R-CHOP. Therefore, the purpose of the present study was to identify factors associated with treatment and survival in a cohort of patients diagnosed with DLBCL during the period when rituximab was introduced, with a particular focus on race/ethnicity.

## Methods

### Data Source

The source of data for this study was the National Cancer Institute's (NCI) SEER cancer registry linked to Medicare enrollment and claims data. This database has been described in detail elsewhere [15]. Briefly, as of 2010, SEER collects and publishes cancer incidence and survival data from 18 population-based cancer registries throughout the United States covering approximately 26% of the US population [16]. SEER coverage includes 23% of African Americans, 40% of Hispanics, 42% of American Indians and Alaska Natives, 53% of Asians, and 70% of Hawaiian/Pacific Islanders.

The registries routinely collect data on patient demographics, primary tumor site, tumor morphology and stage at diagnosis, first course of treatment, and follow-up for vital status. In the SEER-Medicare data, cancer registry data are linked to Medicare enrollment and claims data. For persons age 65 years or older, 97% are eligible for Medicare, and 93% of patients in the SEER files are matched to the Medicare enrollment file [17]. Almost all Medicare beneficiaries have Part A coverage, which includes hospital, skilled nursing facility, hospice, and some home health care, and 96% of Part A beneficiaries choose to enroll in Part B of Medicare, which covers physician and outpatient services. At the time this study was performed, the SEER-Medicare linkage included all Medicare-eligible persons appearing in the SEER data through 2005 and their Medicare claims through 2007.

### Patient Eligibility

Patients were included in this study if they were diagnosed with DLBCL between January 1, 2001 and December 31, 2005, and DLBCL was the first primary cancer diagnosed. Identification of DLBCL was made using two World Health Organization (WHO) International Classification of Diseases for Oncology, 3^rd ^Edition (ICD-O-3) histology codes: 9680 (malignant lymphoma, large B-cell, diffuse, centroblastic, NOS) and 9684 (malignant lymphoma, large B-cell, diffuse, immunoblastic, NOS) [18,19]. Patients were excluded for the following reasons: DLBCL diagnosed before age 65; diagnosis made by death certificate or autopsy; death within the first month following diagnosis; or Medicare enrollment less than 12 months before diagnosis. In addition, to ensure complete claims history, patients had to have been enrolled in both Medicare Parts A and B, with no health maintenance organization (HMO) coverage for 12 months prior to diagnosis. SEER reports date of diagnosis as month and year only. In this study, the first day of the SEER month of diagnosis was assigned as the day of diagnosis. After diagnosis, patients were followed until death, enrollment in an HMO, development of a second primary tumor, or the last date for which Medicare claims were available.

### Treatments

Medicare claims were used to identify the time of the first infused C-I therapy and radiation treatments provided to patients after diagnosis. International Classification of Diseases, 9^th ^Revision, Clinical Modification (ICD-9-CM) procedure codes [20], and Healthcare Common Procedure Coding System (HCPCS) codes [21], and revenue codes were used to identify C-I and radiation therapy from both inpatient and outpatient claims. A list of codes is included in the Appendix.

The first C-I therapy regimen was reconstructed from 30 days of claims after the first C-I therapy infusion. Patients were classified as taking either CHOP or CVP (cyclophosphamide, vincristine, and prednisone) by assuming the use of prednisone when the other agents were present, because oral medications with no intravenous equivalent were not available in SEER-Medicare at the time our study was conducted. The use of rituximab with these was classified as R-CHOP or R-CVP. If other chemotherapy agents were used, or patients had claims indicative of chemotherapy infusions without the HCPCS codes (i.e., J-codes) to identify the specific chemotherapy agents, these were classified as "other" with or without rituximab.

### Mortality and Censoring

The date of death was assigned by using the Medicare date, if available, even in cases where the SEER date was also available. The Medicare date was preferred because it is more current than the SEER date [22]. In cases where the SEER date of death was available but missing for Medicare, the SEER date was used. The cause of death was classified as NHL or other/unknown cause based on ICD-9-CM codes. All other patients were assumed to be alive at the end of the analysis period (December 31, 2007), although they may have been censored earlier for other reasons, such as switching to HMO coverage.

### Patient Characteristics

Patients were described according to their demographic, clinical, and socioeconomic characteristics. Patient age at diagnosis was stratified into four groups: 66 - 69; 70 - 74; 75 - 79; and ≥ 80. Requiring eligible patients to have at least one year of Medicare enrollment prior to diagnosis ensured that the minimum age in the cohort was 66 years. Race/ethnicity was defined using the SEER re-coded race variable in combination with the Medicare race variable as follows: black, if both variables indicated black race; white, if both variables indicated white race, or if the Medicare variable indicated white race and the SEER variable indicated Hispanic ethnicity (since SEER uses Hispanic surname to assign Hispanic ethnicity); and other, which in SEER consists predominantly of American Indian/Native Alaskan, Native Hawaiian or Other Pacific Islander, and Asian [23].

Summary staging is the approach SEER uses to categorize how far a cancer has spread from its point of origin [24]. It uses all information available in the medical record, and is a combination of the most precise clinical and pathological documentation of the extent of disease. DLBCL is classified as Stage I-IV according to the number of lymph node regions, the location of those regions, involvement of the spleen, and involvement of extra-lymphatic organs/sites. With the exception of Stage IV disease, in which all patients have multifocal involvement, patients classified as Stage I-III may or may not have extra-nodal involvement. Consequently, patients also were classified according to whether their disease was confined to one or more lymph node regions (nodal), or involved the spleen or an extra-lymphatic organ or site (extra-nodal). Finally, they were classified according to the histologic subtype of DLBCL: centroblastic (ICD-O-3 code 9680); or immunoblastic (ICD-O-3 code 9684) [18,19].

We used the Medicare inpatient (Part A), outpatient, and physician (Part B) records to calculate an NCI Comorbidity Index score for each patient [25]. This approach [26,27] entails first removing claims that are considered to have unreliable diagnosis coding, such as those for testing procedures used to rule out conditions. Then, remaining diagnosis and procedure codes are used to identify the 15 non-cancer comorbidities in the Charlson Comorbidity Index (CCI) [28]. The algorithms used to identify these conditions reflect the Deyo [29] adaptation of the CCI, and include several procedure codes from the Romano [30] adaptation. A weight is assigned to each condition, and the weights are summed to obtain the index for each patient. Medicare inpatient and outpatient claims, excluding those likely to be made only for testing purposes, were used to identify the presence of anemia, neutropenia, thrombocytopenia, and other cardiovascular disease prior to diagnosis. These are not included in the NCI Comorbidity Index.

Socioeconomic information at the patient level is not available through SEER-Medicare. Instead, the SEER-Medicare dataset contains information from the 2000 Census, reported at the tract level in which the patient lives, for the percent of the population living in poverty and the percent of those age 25 years or older with some college. We used these as indicators of the socioeconomic status of individual patients in the DLBCL cohort. SEER registry (consolidated into 13 regions, with California as a single region, and excluding Arizona Native Americans) and the assigned metropolitan statistical area as recoded by SEER (big metropolitan, metropolitan, urban, less urban, and rural) were used as geographic indicators.

### Statistical Analysis

Cox proportional hazards regression was used to identify factors associated with treatment and survival. Both the time to treatment and survival analyses were stratified by Stage I-II versus III-IV at diagnosis. In addition to time-to-treatment analyses, logistic regression was used to identify factors associated with receiving therapy within 180 days following diagnosis. Survival analysis was conducted with three different mortality endpoints: all-cause mortality; NHL mortality; and other/unknown cause mortality. The base case multivariate survival analyses were performed without treatment as a covariate, such that any racial disparities in access to treatment that also impacted survival were captured in the race covariates in the survival analyses. We then repeated all the multivariate survival analyses with treatment included as a time dependent covariate. We reasoned that comparing the race covariates from models with versus without treatment included would provide insight into the impact racial disparities in access to treatment had on racial disparities in survival.

### Editorial Note

Notification of all NCI approvals for this study was obtained from IMS, Inc., via email correspondence, on July 29th, 2008. At the time this study was approved, NCI did not require Institutional Review Board approval prior to releasing SEER-Medicare data. However, since the SEER-Medicare data contain information about geographic location at the county level, as well as dates of receiving health care services, the SEER-Medicare data are considered by Health Insurance Portability and Accountability Act (HIPAA) requirements as a limited data set, which requires that all investigators sign a Data Use Agreement (DUA) prior to receiving the data. The DUA for this study was executed by Dr. Danese on June 7, 2008.

## Results

We identified 7,048 patients who met the study eligibility criteria (Table 1). The median age at diagnosis was 77 years, 54% were female, 88% were white, and 43% had Stage III or IV disease at diagnosis. Overall, 5,887 (84%) received C-I therapy or radiation treatment during the observation period: 5,555 (94%) received C-I therapy with (1,826: 31%) or without radiation (3,729: 63%); and the remainder (332: 6%) received radiation alone (not shown in Table). Among those who received C-I therapy (5,555), 46% (2,569) received chemotherapy alone, 45% (2,488) received rituximab plus chemotherapy, and the remainder (498:9%) received rituximab alone. R-CHOP was the most common chemotherapy regimen (2,167: 39%).

**Table 1:** Patient Characteristics

		Overall Freq	%	Treatment Freq	%	No Treatment Freq	%	P Value
**Total**		7,048	100	5,887	83.5	1,161	16.5	
**Age**								
	66-69	985	14.0	889	15.1	96	8.3	
	70-74	1,576	22.4	1,384	23.5	192	16.5	< 0.0001
	75-79	1,742	24.7	1,497	25.4	245	21.1	
	≥ 80	2,745	38.9	2,117	36.0	628	54.1	

	Median Age	77	72-82	77	72-82	80	75-86	
								

**Gender**								
	Male	3,228	45.8	2,685	45.6	543	46.8	< 0.01
	Female	3,820	54.2	3,202	54.4	618	53.2	

**Race**								
	Black	240	3.4	171	2.9	59	5.1	
	Other	632	9.0	489	8.3	110	9.5	0.01
	White	6,176	87.6	5,184	88.1	992	85.4	

**Stage at diagnosis**								
	I	2,182	31.0	1,838	31.2	344	29.6	
	II	1,330	18.9	1,158	19.7	172	14.8	
	III	1,009	14.3	866	14.7	143	12.3	< 0.0001
	IV	2,044	28.4	1,661	28.2	383	33.0	
	unknown	483	6.9	364	6.2	119	10.2	

**Histology**							0.0	
	Centroblastic	6,902	97.9	5,773	98.1	1,129	97.2	0.07
	Immunoblastic	146	2.1	114	1.9	32	2.8	

**Lymph node site**								
	Extranodal	4,218	59.8	3,486	59.2	732	63.0	< 0.0001
	Nodal	2,347	33.3	2,037	34.6	310	26.7	
	Unkown	483	6.9	364	6.2	119	10.2	

**NCI Comorbidity Index**								
	0	4,075	57.8	3,458	58.7	617	53.1	< 0.0001
	1	1,729	24.5	1,453	24.7	276	23.8	
	2	707	10.0	579	9.8	128	11.0	
	≥ 3	537	7.6	397	6.7	140	12.1	

**Year of diagnosis**								
	2001	1,300	18.4	1,065	18.1	235	20.2	
	2002	1,395	19.8	1,160	19.7	235	20.2	< 0.01
	2003	1,445	20.5	1,237	21.0	208	17.9	
	2004	1,465	20.8	1,230	20.9	235	20.2	
	2005	1,443	20.5	1,195	20.3	248	21.4	

**SEER region**								
	CT	435	6.2	365	6.2	70	6.0	
	Detroit	482	6.8	409	6.9	73	6.3	
	HI	84	1.2	70	1.2	14	1.2	
	IA	526	7.5	453	7.7	73	6.3	
	NM	166	2.4	133	2.3	33	2.8	
	Seattle	397	5.6	331	5.6	66	5.7	< 0.01
	UT	253	3.6	206	3.5	47	4.0	
	Atlanta	184	2.6	NS	NS	NS	NS	
	Rural GA	20	0.3	NS	NS	NS	NS	
	KY	614	8.7	496	8.4	118	10.2	
	LA	506	7.2	419	7.1	87	7.5	
	NJ	1,223	17.4	1,045	17.8	178	15.3	
	CA	2,158	30.6	1,788	30.4	370	31.9	

**Percent of 25+ year olds in census tract with some college**								0.87
	0-< 25%	2,535	36.0	2,127	36.1	408	35.1	
	≥ 25%	4,513	64.0	3,760	63.9	753	64.9	

**Urban vs.rural**								
	Big Metro	3,867	54.9	3,256	55.3	611	52.6	
	Metro	2,071	29.4	1,708	29.0	363	31.3	0.33
	Urban	410	5.8	342	5.8	68	5.9	
	Less Urban	570	8.1	470	8.0	100	8.6	
	Rural	130	1.8	111	1.9	19	1.6	

**Percent living in poverty in census tract**								
	Missing	38	0.5	NS	NS	NS	NS	
	0-< 5%	2,311	32.8	NS	NS	NS	NS	0.05
	5-< 7%	978	13.9	811	13.8	167	14.4	
	7-< 12%	1,552	22.0	1306	22.2	246	21.2	
	≥ 12%	2,169	30.8	1762	29.9	407	35.1	

**Other conditions**								
	Anemia	652	9.3	501	8.5	151	13.0	< 0.0001
	Neutropenia	14	0.2	NS	NS	NS	NS	0.65
	Thrombocytopenia	43	0.6	NS	NS	NS	NS	0.52
	Cardiovascular disease	849	12.0	659	11.2	190	16.4	< 0.0001
								

Treatment consisted of chemo-immunotherapy, radiation therapy, or both

NS: Not shown due to at least one cell in the characteristic with < 11 observations

**Figure 1:**
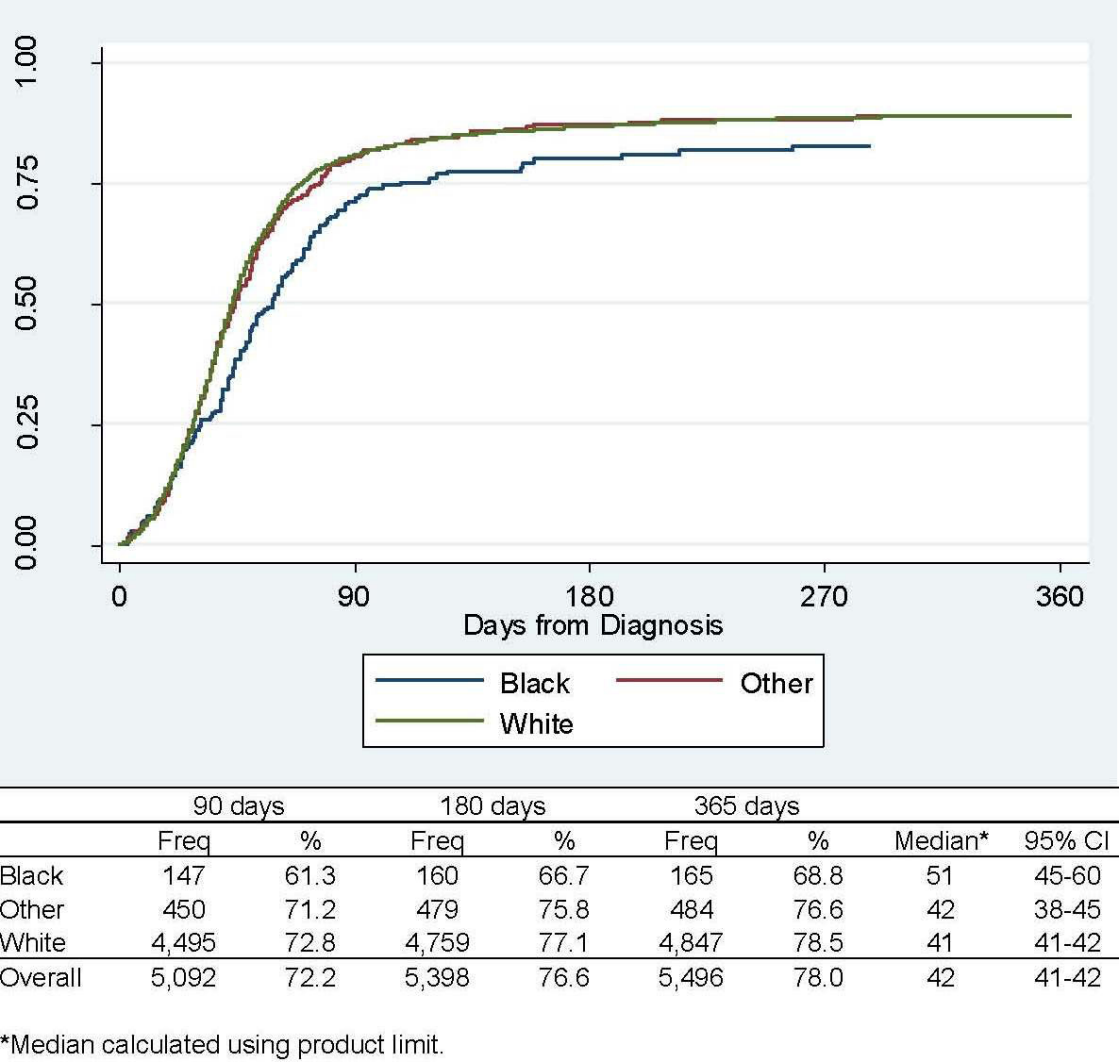
**Time to Treatment, by Race**.

As shown in Figure 1, the median time to first treatment following DLBCL diagnosis was 42 days, and 92% (5,398/5,887) of the patients receiving treatment began within 180 days following diagnosis. The unadjusted time to beginning treatment was 10 days longer for blacks versus whites. In multivariate analysis of time to treatment using Cox regression, the treatment rate was significantly lower among patients ≥ 80 years old, blacks versus whites, those living in a census tract with ≥ 12% poverty, and extra-nodal disease. The treatment rate was higher in those diagnosed with later-stage disease (Table 2). In stratified analyses, the treatment rate was lower in blacks than whites among Stage I-II and among Stage III-IV patients. Findings from the logistic regression analysis of treatment within the first 180 days following treatment were consistent with those from the Cox regression analyses (Table 2), except that generally the effect sizes were larger in the logistic regression analysis. For instance, in the logistic regression model, the OR for treatment among blacks versus whites was 0.63 compared to a HR of 0.77 in the Cox model that included all patients.

**Table 2:** Multivariate Analysis of Treatment

		Time to Treatment - Overall				Time to Treatment - Stage I & II				Time to Treatment - Stage III & IV				Treatment within 180 days			
		**HR**	**p-value**	**95% CI**		**HR**	**p-value**	**95% CI**		**HR**	**p-value**	**95% CI**		**OR**	**p-value**	**95% CI**	
**Age**																	
	**64-< 70**	ref.															
	**70-< 75**	1.00	0.91	0.91	1.08	0.90	0.07	0.79	1.01	1.10	0.16	0.96	1.24	0.73	0.01	0.59	0.91
	**75-< 80**	0.95	0.20	0.87	1.03	0.91	0.12	0.81	1.03	0.99	0.86	0.87	1.12	0.59	< 0.0001	0.48	0.73
	**≥ 80**	0.78	< 0.0001	0.72	0.84	0.69	< 0.0001	0.62	0.77	0.89	0.07	0.79	1.01	0.38	< 0.0001	0.31	0.46

**Gender**																	
	**Female**	ref.															
	**Male**	0.99	0.70	0.94	1.04	1.01	0.83	0.94	1.09	0.97	0.42	0.89	1.05	0.85	0.01	0.76	0.95

**Race**																	
	**White**	ref															
	**Black**	0.77	< 0.001	0.66	0.89	0.76	0.01	0.61	0.95	0.76	0.02	0.60	0.96	0.63	< 0.01	0.47	0.85
	**other**	1.01	0.78	0.92	1.12	1.06	0.42	0.92	1.21	0.95	0.57	0.81	1.12	0.99	0.89	0.79	1.22

**Histology**																	
	**9680**	ref															
	**9684**	0.86	0.10	0.71	1.03	0.70	0.01	0.53	0.92	1.16	0.27	0.89	1.50	0.66	0.03	0.45	0.95

**Stage at diagnosis**																	
	**I**	ref															
	**II**	1.20	< 0.0001	1.11	1.29	1.21	< 0.0001	1.12	1.30					1.32	< 0.01	1.11	1.58
	**III**	1.27	< 0.0001	1.17	1.38									1.04	0.71	0.86	1.26
	**IV**	1.23	< 0.0001	1.14	1.32					0.93	0.37	0.80	1.08	0.91	0.22	0.78	1.06
	**unknown**	0.85	0.01	0.75	0.96									0.56	< 0.0001	0.44	0.71

**NCI Comorbidity Index**																	
	**0**	ref															
	**1**	1.05	0.15	0.98	1.12	1.03	0.53	0.94	1.13	1.09	0.07	0.99	1.20	1.00	0.95	0.86	1.15
	**2**	1.03	0.51	0.94	1.14	1.03	0.64	0.90	1.18	1.09	0.24	0.94	1.26	0.87	0.19	0.71	1.07
	**≥3**	0.93	0.25	0.82	1.05	1.04	0.64	0.88	1.24	0.89	0.25	0.73	1.09	0.56	< 0.0001	0.44	0.71

**Year of diagnosis**																	
	**2001**	ref															
	**2002**	0.99	0.84	0.91	1.08	0.95	0.35	0.84	1.06	1.08	0.25	0.95	1.23	0.95	0.57	0.79	1.14
	**2003**	1.04	0.37	0.96	1.13	1.00	0.99	0.89	1.12	1.10	0.14	0.97	1.25	1.18	0.08	0.98	1.42
	**2004**	1.03	0.53	0.95	1.12	1.00	0.98	0.89	1.12	1.04	0.51	0.92	1.19	1.17	0.09	0.98	1.41
	**2005**	0.99	0.89	0.91	1.08	0.93	0.26	0.83	1.05	1.08	0.24	0.95	1.22	1.00	0.99	0.83	1.20

**Percent of 25+ year olds in census tract with some college**																	
	**0-< 25%**	ref															
	**25-< 50%**	0.95	0.11	0.89	1.01	0.91	0.03	0.83	0.99	0.96	0.39	0.87	1.06	0.92	0.25	0.80	1.06
	**≥50%**	0.84	0.34	0.59	1.20	0.87	0.61	0.52	1.46	0.87	0.68	0.45	1.70	0.50	0.04	0.26	0.98

**Percent living in poverty in census tract**																	
	**0-< 5%**	ref															
	**5-< 7%**	0.92	0.04	0.84	1.00	0.94	0.31	0.84	1.06	0.92	0.18	0.81	1.04	0.87	0.15	0.73	1.05
	**7-< 12%**	0.97	0.36	0.90	1.04	0.95	0.32	0.86	1.05	0.97	0.54	0.86	1.08	0.95	0.51	0.80	1.12
	**≥12%**	0.90	0.01	0.83	0.97	0.92	0.10	0.82	1.02	0.89	0.04	0.79	1.00	0.82	0.02	0.70	0.97

**SEER region**																	
	**CA**	ref															
	**CT**	1.05	0.40	0.93	1.19	1.01	0.87	0.86	1.20	1.10	0.33	0.91	1.32	1.14	0.34	0.87	1.50
	**Detroit**	0.99	0.84	0.89	1.10	0.96	0.57	0.82	1.12	1.09	0.32	0.92	1.28	1.07	0.61	0.83	1.37
	**HI**	0.95	0.65	0.74	1.21	0.90	0.54	0.65	1.25	1.01	0.95	0.68	1.50	1.07	0.81	0.62	1.85
	**IA**	1.16	0.01	1.05	1.29	1.31	< 0.001	1.13	1.51	1.04	0.67	0.88	1.22	1.44	< 0.01	1.12	1.86
	**NM**	1.17	0.09	0.98	1.39	1.23	0.10	0.96	1.58	1.11	0.47	0.84	1.47	0.92	0.68	0.64	1.35
	**Seattle**	1.01	0.93	0.89	1.13	1.08	0.40	0.91	1.28	0.97	0.72	0.82	1.15	1.29	0.07	0.98	1.70
	**UT**	0.93	0.32	0.80	1.08	0.91	0.34	0.75	1.11	0.99	0.90	0.79	1.24	0.90	0.52	0.65	1.24
	**Atlanta**	1.08	0.38	0.91	1.27	1.05	0.71	0.82	1.35	1.09	0.47	0.86	1.40	1.16	0.45	0.79	1.69
	**Rural GA**	1.04	0.89	0.62	1.73	0.76	0.47	0.36	1.61	1.32	0.54	0.54	3.22	0.84	0.72	0.31	2.27
	**KY**	0.95	0.37	0.86	1.06	1.00	0.95	0.87	1.16	0.96	0.58	0.81	1.13	0.91	0.42	0.73	1.14
	**LA**	1.06	0.33	0.95	1.18	1.01	0.88	0.87	1.18	1.18	0.06	0.99	1.41	1.09	0.51	0.85	1.39
	**NJ**	1.05	0.32	0.96	1.14	1.05	0.48	0.93	1.18	1.05	0.51	0.91	1.20	0.96	0.65	0.79	1.16

**Lymph node site**																	
	**Nodal**	ref.															
	**Extra-nodal**	0.90	< 0.01	0.85	0.97	0.90	< 0.01	0.83	0.96	0.95	0.55	0.81	1.12	0.88	0.09	0.75	1.02

**Other conditions**																	
	**Anemia**	1.04	0.42	0.95	1.14	1.04	0.60	0.90	1.19	1.02	0.83	0.88	1.17	0.67	< 0.0001	0.55	0.80
	**Neutropenia**	1.03	0.93	0.56	1.87	0.47	0.19	0.15	1.46	1.51	0.38	0.61	3.75	0.54	0.28	0.18	1.65
	**Thrombocytopenia**	1.35	0.07	0.97	1.88	1.88	0.02	1.12	3.17	0.97	0.88	0.61	1.54	0.51	0.04	0.27	0.97
	**Cardiovascular disease**	0.99	0.77	0.89	1.09	1.01	0.86	0.88	1.16	0.93	0.33	0.79	1.08	0.93	0.49	0.76	1.14

HR: Hazard Ratio

OR: Odds Ratio

There were 4,188 (59% of the population) deaths during the observation period: 2,366 (56%) had NHL listed as the cause of death, and the remaining 1,822 (44%) had another cause listed (919: 50%) or the cause of death was not recorded (903: 50%). The median survival was two years, and 95% survived at least six weeks following diagnosis, which, as reported above, was also the median time to initial treatment. In multivariate survival analysis using Cox regression, which did not include treatment as a covariate, older age, male gender, black race, immunoblastic histology, advanced stage at diagnosis, higher NCI Comorbidity Index, anemia, cardiovascular disease, and living in a census area with ≥ 12% poverty all were associated with higher all-cause mortality (Table 3). Being diagnosed later in the observation period was associated with lower all-cause mortality. Black race was associated with higher mortality due to other/unknown causes but it was not associated with higher mortality due to NHL. In the NHL mortality model, the effect sizes were larger for histology, Stage, year of diagnosis, and anemia than in the other/unknown cause mortality model. In the other/unknown cause mortality model, effect sizes were larger for age, NCI Comorbidity Index, cardiovascular disease, and poverty.

**Table 3:** Multivariate Survival Analysis

		All Cause (4,188 deaths)				NHL (2,366 deaths)				Other/Unknown Cause (1,822 deaths)			
		HR	p-value	95% CI		HR	p-value	95% CI		HR	p-value	95% CI	
**Age**													
	66-< 70	ref.											
	70-< 75	1.38	< 0.0001	1.22	1.55	1.37	< 0.001	1.16	1.61	1.38	< 0.001	1.15	1.66
	75-< 80	1.75	< 0.0001	1.55	1.96	1.75	< 0.0001	1.50	2.05	1.72	< 0.0001	1.44	2.05
	≥ 80	2.69	< 0.0001	2.41	3.00	2.60	< 0.0001	2.24	3.01	2.79	< 0.0001	2.36	3.29

**Gender**													
	Female	ref.											
	Male	1.09	< 0.01	1.03	1.16	1.05	0.22	0.97	1.14	1.14	0.01	1.04	1.25

**Race**													
	White	ref.											
	Black	1.24	0.01	1.06	1.46	1.16	0.19	0.93	1.45	1.35	0.01	1.06	1.71
	Other	1.09	0.17	0.97	1.22	1.11	0.19	0.95	1.30	1.06	0.52	0.89	1.26

**DLBCL histology**													
	9680	ref.											
	9684	1.47	< 0.001	1.21	1.79	1.52	< 0.001	1.19	1.94	1.32	0.11	0.94	1.84

**Stage at diagnosis**													
	I	ref.											
	II	1.27	< 0.0001	1.16	1.40	1.42	< 0.0001	1.25	1.62	1.15	0.05	1.00	1.31
	III	1.51	< 0.0001	1.36	1.68	1.77	< 0.0001	1.53	2.04	1.28	< 0.01	1.10	1.50
	IV	1.86	< 0.0001	1.70	2.02	2.43	< 0.0001	2.16	2.74	1.30	< 0.0001	1.14	1.48
	unknown	1.56	< 0.0001	1.36	1.79	1.45	< 0.001	1.19	1.77	1.72	< 0.0001	1.42	2.08

**NCI Comorbidity Index**													
	0	ref.											
	1	1.19	< 0.0001	1.10	1.28	1.14	0.01	1.03	1.26	1.27	< 0.0001	1.13	1.42
	2	1.44	< 0.0001	1.29	1.60	1.34	< 0.0001	1.16	1.54	1.58	< 0.0001	1.35	1.85
	≥3	1.88	< 0.0001	1.66	2.13	1.70	< 0.0001	1.44	2.01	2.16	< 0.0001	1.79	2.60

**Year of Diagnosis**													
	2001	ref.											
	2002	0.87	< 0.01	0.79	0.96	0.87	0.02	0.77	0.98	0.89	0.14	0.76	1.04
	2003	0.82	< 0.0001	0.74	0.90	0.76	< 0.0001	0.67	0.85	0.95	0.52	0.81	1.11
	2004	0.84	< 0.001	0.76	0.92	0.70	< 0.0001	0.62	0.79	1.16	0.07	0.99	1.35
	2005	0.80	< 0.0001	0.73	0.89	0.39	< 0.0001	0.33	0.45	1.90	< 0.0001	1.63	2.20

**Percent of 25+ year olds in census tract with some college**													
	0-< 25%	ref.											
	25-< 50%	1.00	0.91	0.93	1.07	1.01	0.86	0.91	1.11	0.98	0.77	0.88	1.10
	≥50%	0.90	0.61	0.60	1.35	0.81	0.46	0.45	1.43	1.00	1.00	0.57	1.76

**Percent living in poverty in census tract**													
	0-< 5%	ref.											
	5-< 7%	1.04	0.49	0.94	1.15	1.11	0.13	0.97	1.26	0.96	0.57	0.82	1.12
	7-< 12%	1.03	0.58	0.94	1.12	1.01	0.83	0.90	1.14	1.05	0.51	0.92	1.20
	≥12%	1.13	0.01	1.03	1.23	1.11	0.09	0.99	1.25	1.17	0.03	1.02	1.33

**SEER region**													
	CA	ref.											
	CT	1.08	0.29	0.94	1.25	1.08	0.42	0.89	1.31	1.09	0.45	0.88	1.34
	Detroit	1.12	0.10	0.98	1.27	1.16	0.09	0.98	1.38	1.04	0.72	0.85	1.27
	HI	0.81	0.16	0.60	1.09	0.66	0.07	0.42	1.03	0.98	0.90	0.65	1.47
	IA	0.98	0.81	0.86	1.12	1.08	0.36	0.91	1.28	0.85	0.13	0.70	1.05
	NM	1.24	0.04	1.01	1.52	1.32	0.05	1.00	1.74	1.15	0.38	0.84	1.58
	Seattle	1.02	0.82	0.88	1.17	1.15	0.12	0.96	1.38	0.82	0.09	0.64	1.03
	UT	1.01	0.88	0.85	1.21	1.15	0.24	0.91	1.44	0.84	0.25	0.63	1.13
	Atlanta	0.96	0.71	0.78	1.18	0.98	0.86	0.74	1.28	0.97	0.85	0.72	1.32
	Rural GA	0.97	0.91	0.55	1.72	0.90	0.79	0.40	2.02	1.01	0.99	0.45	2.27
	KY	1.12	0.07	0.99	1.26	1.16	0.07	0.99	1.36	1.06	0.52	0.89	1.27
	LA	1.07	0.34	0.93	1.22	1.13	0.16	0.95	1.35	0.97	0.80	0.80	1.19
	NJ	1.12	0.03	1.01	1.25	1.11	0.15	0.96	1.27	1.16	0.06	1.00	1.35

**Lymph node site**													
	Nodal	ref.											
	Extranodal	1.09	0.05	1.00	1.18	1.06	0.33	0.94	1.19	1.13	0.05	1.00	1.28

**Other conditions**													
	Anemia	1.31	< 0.0001	1.19	1.45	1.37	< 0.0001	1.20	1.55	1.23	0.01	1.05	1.43
	Neutropenia	1.03	0.93	0.55	1.93	0.80	0.67	0.30	2.17	1.35	0.48	0.59	3.08
	Thrombocytopenia	1.03	0.86	0.73	1.46	0.98	0.91	0.62	1.54	1.17	0.57	0.68	2.00
	Cardiovascular disease	1.26	< 0.0001	1.14	1.39	1.09	0.21	0.95	1.26	1.53	< 0.0001	1.31	1.77

HR: Hazard Ratio

In multivariate analyses stratified by Stage I-II and III-IV (Table 4), which did not include treatment as a covariate, black race was associated with significantly higher all-cause mortality among Stage III-IV patients, but not among Stage I-II patients. The effect sizes for age, gender, NCI Comorbidity Index, and cardiovascular disease were larger in the model of Stage I-II patients than the model of Stage III-IV patients, whereas the effect size for year of diagnosis was larger in the model of Stage III-IV patients.

**Table 4:** Multivariate Survival Analysis - All-Cause Mortality, by Stage

		Stage I-II				Stage III-IV			
		HR	p-value	95% CI		HR	p-value	95% CI	
**Age**									
	66-< 70	ref				ref			
	70-< 75	1.40	< 0.001	1.16	1.70	1.32	< 0.01	1.12	1.55
	75-< 80	1.72	< 0.0001	1.43	2.07	1.74	< 0.0001	1.48	2.04
	≥ 80	2.90	< 0.0001	2.44	3.45	2.45	< 0.0001	2.11	2.85

**Gender**									
	Female	ref.							
	Male	1.12	0.02	1.02	1.23	1.06	0.17	0.97	1.16

**Race**									
	White	ref.							
	Black	1.16	0.24	0.91	1.49	1.35	0.01	1.08	1.69
	Other	0.99	0.93	0.83	1.19	1.20	0.04	1.01	1.42

**DLBCL histology**									
	9680	ref.							
	9684	1.45	0.02	1.07	1.96	1.39	0.02	1.06	1.83

**Stage at diagnosis**									
	I	ref.				N/A			
	II	1.29	< 0.0001	1.17	1.41	N/A			
	III	N/A				ref.			
	IV	N/A				1.38	< 0.001	1.16	1.66

**NCI Comorbidity Index**									
	0	ref.							
	1	1.23	< 0.001	1.10	1.38	1.13	0.03	1.01	1.26
	2	1.47	< 0.0001	1.25	1.73	1.38	< 0.0001	1.18	1.61
	≥3	1.99	< 0.0001	1.65	2.39	1.73	< 0.0001	1.44	2.08

**Year of diagnosis**									
	2001	ref.							
	2002	0.94	0.40	0.82	1.08	0.80	< 0.01	0.70	0.91
	2003	0.86	0.04	0.75	1.00	0.78	< 0.001	0.68	0.89
	2004	0.88	0.10	0.76	1.02	0.77	< 0.001	0.67	0.88
	2005	0.88	0.09	0.75	1.02	0.75	< 0.0001	0.65	0.86

**Percent of 25+ year olds in census tract with some college**									
	0-< 25%	ref.							
	25-< 50%	1.04	0.47	0.93	1.16	0.97	0.56	0.87	1.08
	≥50%	0.54	0.09	0.26	1.09	1.28	0.45	0.68	2.42

**Percent living in poverty in census tract**									
	0-< 5%	ref.							
	5-< 7%	1.06	0.43	0.92	1.23	1.02	0.78	0.88	1.18
	7-< 12%	1.00	1.00	0.87	1.14	1.09	0.18	0.96	1.24
	≥12%	1.21	< 0.01	1.06	1.39	1.12	0.09	0.98	1.27

**SEER region**									
	CA	ref.							
	CT	1.16	0.15	0.95	1.42	1.04	0.75	0.84	1.28
	Detroit	1.10	0.35	0.90	1.33	1.19	0.07	0.99	1.43
	HI	0.71	0.13	0.46	1.11	0.87	0.53	0.57	1.34
	IA	0.91	0.38	0.75	1.12	1.01	0.95	0.84	1.21
	NM	1.09	0.61	0.79	1.49	1.27	0.13	0.93	1.73
	Seattle	0.98	0.87	0.78	1.23	1.03	0.75	0.85	1.25
	UT	1.04	0.75	0.81	1.34	1.01	0.94	0.78	1.31
	Atlanta	1.28	0.12	0.94	1.74	0.82	0.19	0.62	1.10
	Rural GA	0.93	0.86	0.41	2.10	1.09	0.87	0.40	2.96
	KY	1.01	0.91	0.84	1.21	1.27	0.01	1.06	1.51
	LA	1.05	0.65	0.86	1.27	1.06	0.54	0.88	1.29
	NJ	1.26	< 0.01	1.08	1.47	1.04	0.60	0.90	1.21

**Lymph node site**									
	Nodal	ref.							
	Extranodal	1.13	0.01	1.03	1.24	0.94	0.52	0.77	1.14

**Other conditions**									
	Anemia	1.37	< 0.0001	1.18	1.60	1.25	< 0.01	1.08	1.44
	Neutropenia	1.53	0.35	0.62	3.77	0.59	0.37	0.19	1.87
	Thrombocytopenia	1.23	0.49	0.69	2.19	0.96	0.87	0.61	1.53
	Cardiovascular disease	1.33	< 0.001	1.14	1.54	1.18	0.04	1.01	1.37

HR: Hazard Ratio

N/A: Not Applicable

In multivariate survival analyses that did include treatment (Yes/No) as a covariate, the associations between black race and all-cause mortality, and between black race and other/unknown cause mortality, remained statistically significant (HR = 1.19, P = 0.04; and HR 1.27, P = 0.05, respectively). In the multivariate analyses stratified by Stage, black race remained significant for patients diagnosed with Stage III-IV disease (HR = 1.28, P = 0.03). Treatment was statistically significant in all five models.

## Discussion

We conducted a study using SEER-Medicare to identify factors associated with treatment and mortality in an elderly cohort of patients diagnosed with DLBCL. In particular, we sought to understand whether racial differences in treatment observed in an earlier study of SEER-Medicare data [14] were present in more recent SEER-Medicare data. Also, we reasoned that any observed differences in treatment might now translate into greater differences in survival following the introduction of rituximab, which, when added to CHOP, has been shown to improve overall survival in aggressive NHL [6-10]. Our findings show that blacks were less likely to receive treatment than whites. In multivariate analysis of time to initial treatment, the adjusted rate of treatment was 23% lower in blacks than whites. Furthermore, blacks were 37% less likely than whites to begin treatment within the first 180 days following diagnosis. The difference between the black race coefficients in these two models, as well as the Kaplan-Meier curves illustrating time to first treatment, suggest that while there were persistent differences in treatment rates during the entire observation period, the differences were greatest within the first six months following diagnosis. This is of considerable concern in DLBCL since it is an aggressive type of NHL where frontline treatment with C-I therapy is now recommended in most instances [3].

We next examined factors associated with mortality. In the multivariate survival analysis, black race was associated with 24% higher all-cause mortality, adjusting for demographic and clinical covariables. When we divided the cause of death into that recorded as NHL versus other/unknown, we found that black race was associated with 35% higher mortality due to other/unknown causes. However, black race was not associated with statistically significantly higher NHL mortality. When we compared these two models, we found that several of the cancer covariables, e.g. Stage, histology, and anemia, had larger effects in the NHL mortality model than in the other/unknown cause mortality model. Also, the effect of year of diagnosis was greater in the NHL model than in the other/unknown cause mortality model, perhaps reflecting improvements in treatment of DLBCL over time. In contrast, several of the demographic and general comorbidity variables, e.g. gender, NCI Comorbidity Index, poverty, and cardiovascular disease, had larger effects in the other/unknown cause mortality model than the NHL model. When we stratified the analysis of all-cause mortality by disease Stage at diagnosis, we found that black race was associated with 35% higher mortality in Stage III-IV patients, but not with statistically significantly higher mortality in Stage I-II patients. In general, the cancer covariables had greater effects in the Stage III-IV model than in the Stage I-II model, and the opposite was true for the demographic and general comorbidity variables. It is interesting to note that when treatment was added as a covariate to the survival models, the results pertaining to black race were consistent with those in the base case analyses, except that the coefficients were smaller in the models that included treatment. This suggests that poorer access to treatment partially, but not fully, explains the disparities in survival we observed.

While our findings suggest that racial differences in all-cause mortality are due primarily to causes other than NHL, it is important to interpret the findings in the context of several limitations of our study. First, in SEER, the cause of death is obtained from state death certificates, and the underlying cause as coded by state health departments is accepted [22]. There is no cause of death listed on the Medicare side of SEER-Medicare, and it is therefore acknowledged that cause of death is inherently less reliable than other SEER variables [22]. In our study, 50% of patients who died from other/unknown causes had no cause of death assigned. It is possible that some of these patients died of causes related to NHL. This might have artificially inflated the actual association between black race and other/unknown cause mortality. We are not aware of any studies that validate the cause of death as recorded in SEER for patients with NHL. However, a recent study showed that cause of death coding for colon cancer in SEER had an estimated validity of 94.6% [31]. Of note, the estimated validity was lower for blacks (84.4%) compared to whites (95.4%), suggesting that any misclassification in our study could have impacted black patients disproportionately. If fewer blacks than whites were correctly assigned NHL as the cause of death, this could have artificially deflated the actual association between black race and NHL mortality in our analysis.

Second, observed disparities in cancer outcomes among racial and ethnic minorities can reflect obstacles to receiving health care services, including prevention, early detection, and high quality treatment [1]. Although one overriding factor is poverty [1], disparities have been shown to exist even when access-related factors such as insurance status and the ability to pay for care are the same across racial and ethnic groups [11]. Our study was conducted in a cohort of SEER-Medicare patients who had Medicare Part A and Part B insurance for at least one year before DLBCL diagnosis and throughout the observation period following diagnosis. Furthermore, we included measures of income and education in our multivariate treatment and survival models. Patient selection and the inclusion of income and education variables should have reduced the impact of insurance status and ability to pay as access-related factors in our analyses. However, we were unable to account for potential differences in Part B and Part D supplemental insurance (to cover Part B copayments) in our analyses. Also, we were unable to account for disparities in health insurance prior to Medicare eligibility, which could have affected access to health services, comorbidity, and severity of DLBCL at diagnosis in ways we could not adjust for in our analyses. Moreover, since SEER-Medicare does not report income and education levels for individual patients, we relied on census tract-level data.

Third, our study was based on the same data set used by Wang and colleagues in their earlier study [14], and our approaches were similar. Although both studies showed racial disparities in treatment, only ours also showed a difference in mortality. However, since we did not have access to SEER-Medicare data for the earlier time period in which Wang and colleagues conducted their study, we cannot conclude that the racial disparities in mortality we observed indicate a fundamental change from the earlier period. Rather, it could reflect differences in the patient population or analytic approach between our study and that of Wang and colleagues [14].

Finally, our study included a cohort of very elderly patients, with a median age of 77 years. As shown, mortality was high in this population, with only 50% surviving beyond two years. As a result, our findings may not be applicable to younger patients who have a lower risk of mortality overall. Certainly, studies of current treatment modalities for DLBCL would suggest survival is considerably better in younger patients [6].

## Conclusions

Our findings show that the treatment rate was lower and the mortality rate was higher in black compared to white patients diagnosed with DLBCL. It is likely that the observed differences in mortality between blacks and whites are due to a number of factors, including differences in cancer and non-cancer related morbidity, as well as differences in treatment.

## Competing interests

This work was funded by Genentech, Inc. Drs. Danese, Griffiths, Gleeson, and Knopf are consultants to Genentech, Inc.

## Authors' contributions

RG, MG, KK, and MD participated in the design of the study. MG performed the statistical analyses. RG, MG, KK, and MD reviewed the results, participated in writing the manuscript, and reviewed the final manuscript.

## Appendix

**Table 5:** Codes for Identifying Chemo-Immunotherapy and Radiation from Medicare Inpatient and Outpatient Claims

Specific Agents	HCPCS
Fludarabine	J9185
Cyclophosphamide	J8530 J9070 J9080 J9090-J9097
Vincristine	J9370 J9375 J9380
Rituximab	J9310
Mitoxantrone	J9293
Doxorubicin	J9001 J9000
Bleomycin	J9040
Alemtuzumab	J9010
Cisplatin	J9060 J9062
Etoposide	J8560 J9181 J9182
Cytarabine	J9098 J9100 J9110
Pentostatin	J9268
Gemcitabine	J9201
Interferon alpha	J9212 J9213 J9214
Denileukin Difitox	J9160
Methotrexate	J9250 J9260 J8610
Mesna	J9290
Leucovorin	J9064

**Other**	
HCPCS J Codes	J9xxxx (other than above)
HCPCS (other)	964xx or 965xx (other than above) Q0083 Q0084 Q0085
ICD-9-CM	
diagnosis	V581,V662,V672
DRG	410
ICD-9-CM	
Procedure	ICD-9 9925
Revenue Center	0331,0332,0335

**Radiation Codes**	

HCPCS	77401-77499 77750-77799
Diagnosis codes	ICD-9 V580 V661 V671
Procedure codes	ICD-9 9921 9222 9223 9224 9225 9226 9227 9228 9229

HCPCS - Healthcare Common Procedure Coding System

ICD-9 - International Classification of Diseases, 9^th ^Revision, Clinical Modification DRG - Diagnosis-Related Group

## Pre-publication history

The pre-publication history for this paper can be accessed here:

http://www.biomedcentral.com/1471-2407/10/625/prepub
